# Some Suggestions on Bridge-Work

**Published:** 1896-09

**Authors:** Frank L. Platt

**Affiliations:** San Francisco.


ARTICLE II.

### SOME SUGGESTIONS ON BRIDGE-WORK.

BY FRANK L. PLATT, D. D. S., SAN FRANCISCO.

The day is fast approaching when the dentist whose ability limits the sphere of his usefulness to only the ordinary operations of dental surgery, who seldom constructs a tooth-crown of any kind, and rarely ever ventures to insert a piece of bridge-work, will find himself one of the remaining members of an obsolete school; a man behind the times in which he lives; who must reform his ways and develop the latest talent he may possess or suddenly find his occupation gone. He must grasp and develop new methods and ideas, and learn to apply them in the way best suited to his individual necessities.

Conservatism may act as an excellent brake on the too rapid progress of reckless experimentation, but, standing alone, can only effectually clog the progress of any liberal art or science.

While we, as dentists, have made great progress in the past and are destined, as we advance in higher education, to still greater accomplishments, there still remains in our ranks a large number of those, who, to hide their own ignorance perhaps, decry new inventions as being harmful and worthless; not even giving them the test of experiment.

Bridge-work has suffered much in this direction, and that there is a lamentable ingorance prevalent in regard to its qualities, construction and usefulness, both in the profession and laity, is evident from the experience of all of us. How often do we have to tell our patients what bridge-work is, and answer such questions as, "Well, Doctor, will not it cause the teeth around it to decay? Will not it wear out the teeth to which it is fastened? Can it be taken out and cleaned?" etc., almost without end. We have many of us seen pieces of bridge-work, said to have been made and inserted by men of good repute and supposed ability, which seemed to embody in a single case nearly all the evils accredited to bridge-work in general and poor bridgework in particular.

Such experiences show too plainly the need of more general education in the higher branches of our art, and, as far as the public is concerned, emphasize the fact that dentists, as instructors, are not doing—either from lack of ability or interest—all that is required of them by their patients and fellow practitioners. To the progressive, up-to-date dentist there is probably no part of his work more interesting, more satisfactory when well executed, or requiring more exact mechanical ability, than the construction of bridge-work.

While it is highly probable that porcelain bridge-work will before many years entirely supplant that now most in vogue, it is now so nearly in its infancy and so little understood that this paper will not touch upon it, but will be confined to gold and porcelain bridge-work of ordinary construction. To be successful in this art the practitioner must possess a nice sense of discrimination in regard to the general fitness of things, sufficient interest and enthusiasm in his work to call forth his best efforts in each and every case; good judgment in regard to the requirements of the case on hand, and, above all, a sterling integrity, which will effectually prevent his recommendation of any form of dental appliance he does not believe to be fully adapted to the case under consideration, or give satisfaction when completed, both to himself and his patient.

A good old dentist, whose sayings are familiar to many of us, has said that "where gold is indicated there is no better filling material than gold; and where amalgam is indicated there is no better filling material than amalgam." So we may say with equal truth, that where bridge-work is indicated there is no better form of artificial denture than bridge-work.

The question, then, naturally arises "Where is bridge-work indicated?" Briefly, it is indicated wherever it will do comfortable and acceptable service, and present a reasonably pleasing appearance.

It is not indicated, however, in every case where a patient thinks he wants it, or where the operator can get a good fat fee for making it, regardless of future consequences. One of the strongest points urged against bridge-work is its too frequent uncleanliness, some writers, even claiming that they can detect the presence of bridge-work in the mouth of a patient as soon as he enters the office. Making all due allowance for the extraordinary development of the olfactory sense of such a dentist, and sympathizing with him for possessing it, we must admit that there is some just ground for this familiar criticism.

Too many bridges are supported by ill-fitting crowns, and so clumsily put together as to accumulate and hold in offensive masses a considerable quantity of food and tobacco. Too many bridges are placed where plates should be worn, and too many people are extremely careless in the care and preservation of their teeth, be they natural or artificial; and it is not fair or just to condemn all bridge-work because one piece fails, or to say all of it is dirty because some of it is not clean.

It has never been my good fortune, as yet, to see a piece of removable bridge-work which was either clean or substantial; and as it seems that the main object of bridge-work is to replace the lost natural teeth with a denture which will, as far as possible, be their counterpart in stability and usefulness, a movable bridge cannot so far fulfill the requirements of the case as one immovable, clean and strong.

No honest civil engineer will construct a bridge where he cannot get solid foundations for his piers, or where the channel beneath the bridge cannot be kept reasonably free from obstructions, or the accumulation of waste-matter, that may eventually cause the undermining and destruction of the whole structure. So too, no honest dentist will endeavor to fasten a piece of bridge-work on weak or diseased teeth or roots, or on teeth which do not so stand in relation to each other as to bear the additional strain put upon them, and allow a reasonably perfect adaptation of the crowns which support the bridge to the teeth upon which they are placed.

A bridge should never be inserted when the span is so great that in order to make it bear the strain of mastication it must be made heavy, clumsy, and of such a form as to be decidedly unclean.

Such, for example, are fixed bridges, depending in part for support on saddle-shaped plates resting on the gum, or those having too heavy and wide a bar of solder approaching too near the gum between the supports of the bridge. Neither should two or more portions of the same bridge be fastened together by a band passing around a natural unprotected tooth, or by a band passing behind or over it. Such contrivances cannot be kept clean, and will sooner or later lead to the decay and destruction of the teeth around which they are placed.

The same argument will generally hold good against the custom of supporting bridges by spuds or bars anchored into either gold or amalgam fillings. The space beneath the bar or spud cannot or will not be kept clean, and the filling will fail at the cervical border, or else will be wholly loosened by the strain put upon it in mastication. There is also a danger of splitting bicuspid teeth by this method of supporting bridges.

It is a better plan to fasten the bridge in place by means of gold or Richmond crowns, or a combination of both, than to depend too much on the stability of a filling which may do well if it alone remains intact and preserves the tooth in which it is placed.

So, also, too much dependence must not be placed upon open-faced gold crowns, and they should not be used upon short, weak teeth, or those already containing larger approximate fillings, rendering them liable at any time to recurrence of decay.

Considering the present state of the art of implanting teeth, and the fact that such teeth in many cases serve only a comparatively temporary purpose, it seems hardly advisable to make general use of them for the support of bridge-work. If, however, a patient will be content to take chances on the durability of the work, and cannot wear a plate comfortably and serviceably, it may be advisable to try the experiment of implantation. Most of our patients, however, look upon such work as an expensive luxury, which, when obtained, should last at least a fair share o the balance of their lives; and until such a time as the general public can be educated to look with favor upon work which lasts only from one to four years, the general use of implanted teeth in crown and bridge-work will not meet with very much approval. Where implantation is a success it serves a most useful purpose, and will always rank as one of the leading discoveries in dental procedure of the present century. But, until its principles are more fully understood, and the absorption of the roots of implanted teeth is effectually prevented, it cannot be looked upon as coming fully up to the standard of durability now ordinarily demanded in dental operations.

The points, then, to be kept most in mind in the construction of bridge-work may be briefly summed up as follows:

Never place a gold crown or band upon a tooth or root until said tooth or root has been so trimmed and shaped that the cap or band will fit *tightly* and protect *thoroughly* the cervical border of the tooth on which it is placed.

Never try to bridge too long a span—as, for instance, from central to lateral incisor, to second or third molar, or from bicuspid to bicuspid, with no intermediate support. The leverage in such cases is invariably too great, and the loss of both bridge and supports only a matter of a little time.

Always protect the facings or plate-teeth used, with heavy cutting edges or cusps of solid solder and gold. To do less than this, even for the sake of appearances, only invites the breaking of the facings, and the unpleasant task of removing and repairing the bridge.

Always finish the work thoroughly, so it may the more easily be kept clean.

Finally, *do honest work*, and do it in the best manner that your ability will permit.—*Pacific Stomatological Gazette.*

